# Editorial: Deciphering population neuronal dynamics: from theories to experiments

**DOI:** 10.3389/fnsys.2023.1193488

**Published:** 2023-04-20

**Authors:** Hongdian Yang, Woodrow L. Shew, Shan Yu, Artur Luczak, Carsen Stringer, Michael Okun

**Affiliations:** ^1^Department of Molecular, Cell and Systems Biology, University of California, Riverside, Riverside, CA, United States; ^2^Department of Physics, University of Arkansas, Fayetteville, AR, United States; ^3^Brainnetome Center and National Laboratory of Pattern Recognition, Institute of Automation, Chinese Academy of Sciences, Beijing, China; ^4^Department of Neuroscience, Canadian Center for Behavioural Neuroscience, University of Lethbridge, Lethbridge, AB, Canada; ^5^Howard Hughes Medical Institute (HHMI) Janelia Research Campus, Ashburn, VA, United States; ^6^Department of Psychology and Neuroscience Institute, University of Sheffield, Sheffield, United Kingdom

**Keywords:** population dynamics, criticality, gamma oscillation, E/I balance, recurrent neural network (RNN)

Animals experience sensory stimuli and exhibit behaviors with many timescales. Therefore, their brains must integrate information across such temporal scales. Groups of neurons also exhibit diverse temporal and spatial scales of coordinated population activity, which may enable multiscale brain function.

Various approaches are used to investigate the many temporal and spatial scales of neuronal population activity, even in the absence of external stimuli. One traditional approach is to examine activity in particular frequency bands which represents synchronized oscillations across the population. For instance, gamma frequency oscillations (30–100 Hz) have been observed in multiple brain regions and have been correlated with processes such as sensory perception (e.g., Womelsdorf et al., 2012) or attention and working memory (e.g., Lundqvist et al., 2018). These oscillations are hypothesized to play an essential role in promoting long-range communication among different brain regions and to facilitate cognitive functions (Fries, 2009).

Another approach to studying multiscale neural activity is motivated by the criticality hypothesis, which posits that a neuronal network poised at a tipping point between order (synchrony) and disorder (asynchrony) exhibits diverse spatiotemporal scales of activity (Plenz et al., 2021; Beggs, 2022). At criticality, temporal and spatial activation distributions have power law scaling. Previous work suggests that the diverse timescales of criticality are directly linked to the diverse timescales of behavior (Stringer et al., 2019; Jones et al., 2023) and integration of information across brain regions (Fagerholm et al., 2016).

Characterizing multiscale population dynamics and studying their mechanisms is crucial to understanding how the brain processes multiscale sensory information and generates multiscale behavior. The articles in this issue report progress toward this goal: they investigate neuronal dynamics, at specific or multiple scales, with and without sensory stimuli, and propose theoretical frameworks for modeling these dynamics.

The work of Mariani et al. examined whether multi-unit spiking events and field potentials in the cortex of anesthetized rats follow power law scaling. Recently it was realized that there are multiple caveats with judging whether such experimental data truly exhibits criticality. The present study takes on board many of the precautions that were proposed. The work concludes that under the investigated conditions the neuronal activity is consistent with the criticality hypothesis. Interestingly, the authors reproduced the recently reported finding that the ratio between spatial and temporal exponents in spiking avalanches is ~1.28 (Fontenele et al., 2019). Additionally, the authors found brief synchronization of the network after an external sensory stimulus. The origins of power law scaling in cortical networks and the modulation of cortical dynamics by sensory stimuli remain open questions for further theoretical work.

What are the network mechanisms that may explain such dynamical activities observed at the levels of individual neurons and neuronal populations? It is known that balanced excitation (E) and inhibition (I) within a network is crucial for efficient information processing. For a single neuron, balanced E/I means that excitatory and inhibitory synaptic inputs closely track each other, leading to irregular firing. At the population level, the E/I balance is usually manifested as stable activity propagation among different neurons, avoiding either run-away or diminishing activities and leading to rich collective behaviors such as avalanche dynamics and oscillations. However, it is unclear how asynchronous firings of individual neurons can coexist with synchronous population activities in an E/I balanced state. Liang et al. built a neuronal network model that exhibits irregular firing, oscillations, and critical avalanche dynamics through changes in only the inhibitory synaptic decay timescale. Importantly, through a mean-field analysis, the authors showed that such an E/I balance is characterized by a stable state associated with a Hopf bifurcation process. These results provide mechanistic insight into how the interaction between excitation and inhibition in a network will determine microscopic and macroscopic dynamics, thereby affecting information processing in the brain.

Liang et al. examined how networks of neurons can exhibit oscillations and other coordinated dynamics. However, properties of individual neurons may also contribute to such dynamics. In particular, Li et al. explored an unusual mechanism that could regulate gamma oscillations, namely the potential role of autapse. The autapse, or auto-synapse, refers to a synaptic connection of a neuron onto itself. Autapses have been found in a number of brain regions, including the cerebral cortex, hippocampus and striatum (e.g., Tamás et al., 1997; Bekkers, 2003), however, their functional roles remain a mystery. By modeling excitatory and inhibitory autapses as excitatory and inhibitory self-feedback loops, the authors conducted simulation and theoretical analyses to explore their influence on gamma oscillations. The authors found that excitatory self-feedback connections promote the generation of gamma oscillation, while excitatory and inhibitory self-feedback connections regulate oscillation frequency in a complementary manner. This work provides new insights into the neuronal basis of gamma oscillation and suggests a functional role for autapses.

The modeling analyses of Li et al. focused primarily on neural activity in the absence of sensory stimuli. However, sensory stimuli also shape neural network dynamics. The work of Hashemnia et al. explored the similarities and differences between the temporal dynamics of human EEG and a recurrent neural network (RNN) model named *Deep Speech* performing the same speech recognition task. It examined whether the success of deep learning methods in recognizing natural human speech could be attributed to their ability to use time-dependent features, similar to the mechanisms observed in humans during speech perception. By presenting identical speech stimuli to both human listeners and the *Deep Speech*, the authors found that units, particularly in the recurrent layer, tracked envelope fluctuations of the speech signal in a similar way to the EEG signals measured at frontocentral electrodes. Furthermore, a Representational Dissimilarity Analysis suggested that both human EEG and *Deep Speech* Clustered similarities between sentence representations in the same way. Uncovering shared informational architectures in biological brains and artificial neural networks is important because it helps us understand the computational principles underlying brain dynamics (Richards et al., 2019; Luczak et al., 2022).

All these studies integrated experimental findings with theoretical modeling insights to provide new knowledge on the structure of neural dynamics. We hope that these results inspire further research of neural dynamics, including new theories and experiments to test them.

## Author contributions

All authors listed have made a substantial, direct, and intellectual contribution to the work and approved it for publication.
